# α-Mangostin inhibits LPS-induced bone resorption by restricting osteoclastogenesis via NF-κB and MAPK signaling

**DOI:** 10.1186/s13020-022-00589-5

**Published:** 2022-03-05

**Authors:** Wenkan Zhang, Guangyao Jiang, Xiaozhong Zhou, Leyi Huang, Jiahong Meng, Bin He, Yiying Qi

**Affiliations:** 1 Department of Orthopedic Surgery, The Second Affiliated Hospital, Zhejiang University School of Medicine, No. 88, Jiefang Road, Hangzhou, 310009 China; 2 Orthopedic Research Institute of Zhejiang University, Hangzhou, China; 3 Department of Orthopedic Surgery, The Fourth Affiliated Hospital, Zhejiang University School of Medicine, Yiwu, 322000 Zhejiang China

**Keywords:** Osteoclast, α-mangostin, NF-κB, MAPK, Osteolysis

## Abstract

**Background:**

Excessive osteoclast activation is an important cause of imbalanced bone remodeling that leads to pathological bone destruction. This is a clear feature of many osteolytic diseases such as rheumatoid arthritis, osteoporosis, and osteolysis around prostheses. Because many natural compounds have therapeutic potential for treating these diseases by suppressing osteoclast formation and function, we hypothesized that α-mangostin, a natural compound isolated from mangosteen, might be a promising treatment as it exhibits anti‐inflammatory, anticancer, and cardioprotective effects.

**Methods:**

We evaluated the therapeutic effect of α-mangostin on the processes of osteoclast formation and bone resorption. The receptor activator of nuclear factor-κB (NF-κB) ligand (RANKL) induces osteoclast formation in vitro, and potential pathways of α-mangostin to inhibit osteoclast differentiation and function were explored. A mouse model of lipopolysaccharide‐induced calvarial osteolysis was established. Subsequently, micro-computed tomography and histological assays were used to evaluate the effect of α-mangostin in preventing inflammatory osteolysis.

**Results:**

We found that α-mangostin could inhibit RANKL-induced osteoclastogenesis and reduced osteoclast‐related gene expression in vitro. F-actin ring immunofluorescence and resorption pit assays indicated that α-mangostin also inhibited osteoclast functions. It achieved these effects by disrupting the activation of NF-κB/mitogen-activated protein kinase signaling pathways. Our in vivo data revealed that α-mangostin could protect mouse calvarial bone from osteolysis.

**Conclusions:**

Our findings demonstrate that α-mangostin can inhibit osteoclastogenesis both in vitro and in vivo and may be a potential option for treating osteoclast-related diseases.

**Supplementary Information:**

The online version contains supplementary material available at 10.1186/s13020-022-00589-5.

## Introduction

Bone remodeling is achieved by an appropriate balance between osteoclastic bone resorption and osteoblastic bone formation, and these important metabolic processes regulate bone structure and function [1]. Osteoclasts originating from hematopoietic monocyte/macrophage precursors are the only known cells that can resorb bone in the human body [2, 3]. Excessive osteoclast activity leads to disproportionate osteoclastic bone resorption, disrupts the balance between bone resorption and bone formation, and causes a variety of bone disorders including osteoporosis, rheumatoid arthritis, periodontal disease, and metastatic cancers [4, 5]. Therefore, inhibiting osteoclast activity might be and effective treatment strategy for such diseases.

Receptor activators of nuclear factor-κB (NF-κB) ligand (RANKL) and macrophage colony-stimulating factor have been shown to mediate osteoclast differentiation by activating different signaling cascades, such as NF-κB and mitogen-activated protein kinase (MAPK) pathways [6–8]. These signaling pathways promote the expression of transcription factors including activator protein‐1 and nuclear factor of activated T cells c1 (NFATc1), which are the key transcription factors for osteoclast differentiation, and finally promote the differentiation and activation of monocyte‐macrophage precursors into osteoclasts [9, 10]. As such, drugs that suppress RANKL-induced signaling have great potential to prevent these osteoclast-related diseases. Here we explored the effect of α-mangostin on RANKL‐induced NF‐κB activation and osteoclastogenesis was explored.

α-mangostin is the most representative xanthone isolated from the pericarp of mangosteen and was reported to have a variety of pharmacological effects [11]. Specifically, α-mangostin has potential usage as an anticancer treatment and can be regarded as a chemopreventive agent for oral cancer, colon cancer, pancreatic cancer, breast cancer, and cutaneous carcinoma [12, 13]. It also has reported anti-inflammatory, anti-bacterial, anti-malarial, and anti-obesity effects action [11, 14–16]. Furthermore, α-mangostin has been shown to improve cardiovascular and digestive system health, as well as controlling free radical oxidation [17–19]. Recent research has shown that α-mangostin can inhibit osteoarthritis (OA) progression by suppressing mitochondrial apoptosis of chondrocytes induced by NF-κB pathway activation [20]. Nevertheless, there are few studies on the effects of α-mangostin on osteoclasts and osteolytic diseases [21]. Based on previous results showing that α-mangostin had potential therapeutic value in the treatment of OA through inhibiting the production of nitric oxide (NO) and prostaglandin E2, as well as interleukin (IL)-1β-induced phosphorylation of the NF-κB signaling proteins, we hypothesized that α-mangostin might be a novel candidate for treatment of osteoclast-related diseases based on its ability to inhibit osteoclastogenesis.

Here we studied the effects of α-mangostin on the induction and outcomes of RANKL-induced osteoclastogenesis, then explored its mechanism and discovered that its affects two key signaling pathways: NF-κB and MAPK. Finally, the bone protective effect of α-mangostin was verified in a lipopolysaccharide (LPS)-induced osteolysis calvarial mouse model.

## Materials and methods

### Media and reagents

**Fig. 1:**
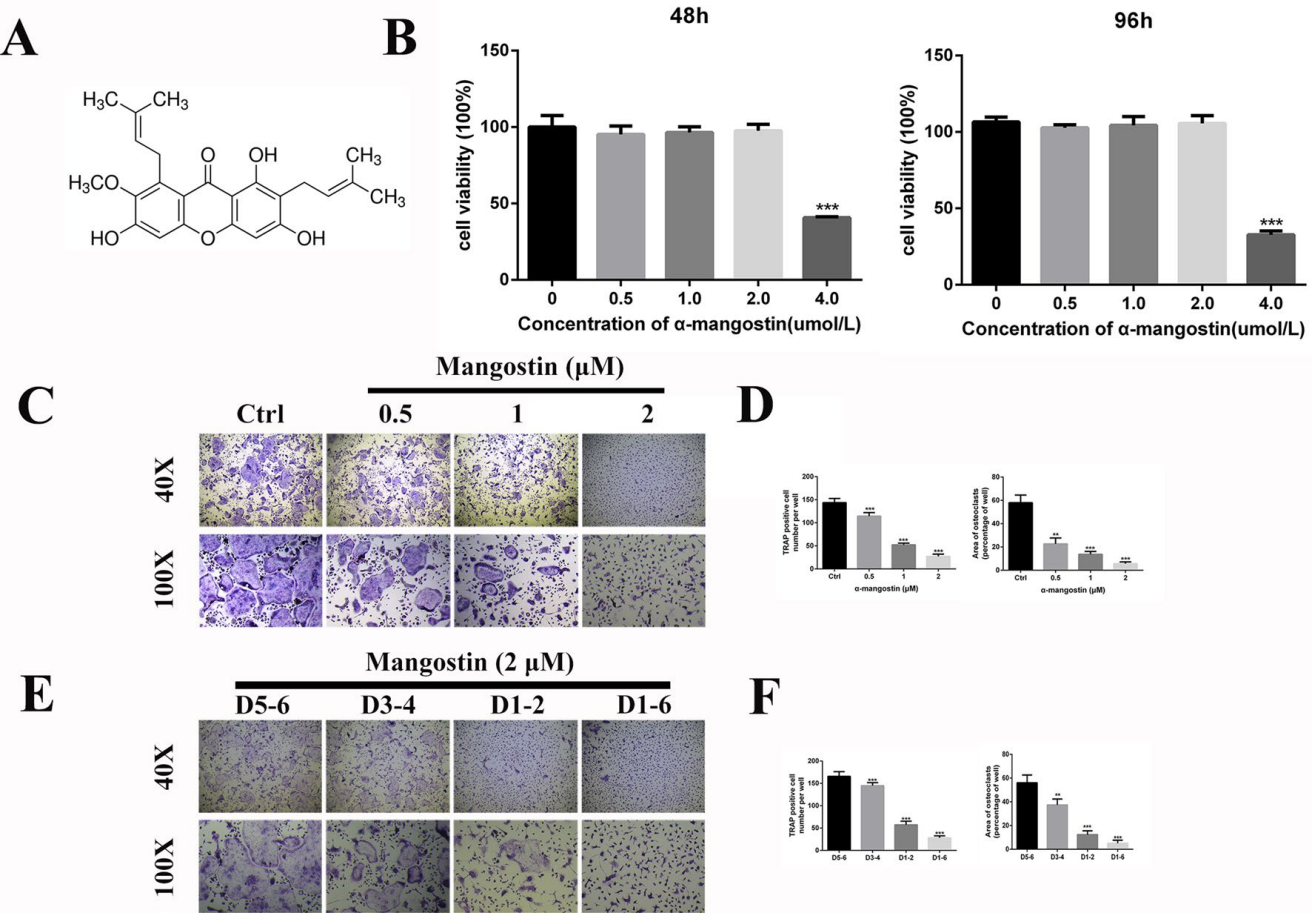
α-mangostin inhibits RANKL‐induced osteoclastogenesis in vitro. **A** The molecular structure of α-mangostin comes from the official website of sigma-aldrich. **B** BMMs were treated with different concentrations of α-mangostin for 48 h or 96 h, and viability of BMMs was measured by CCK-8 assay, N = 3. **C** BMMs were treated with M‐CSF (25 ng/mL) and RANKL (50 ng/mL) in the presence pf the indicated α-mangostin concentrations for 5 days. Cells were then stained for TRAP activity and were photographed. **D** The number and area of TRAP-positive cells were analyzed, N = 3. **E** BMMs were stimulated with M‐CSF and RANKL for 5 days, and 2 μmol/L α-mangostin was added at different stages. Scale bars:200 µm (upper layer of **C**, **E**), 500 µm (Lower layer of **C**, **E**). **F** The number and area of TRAP-positive cells were analyzed, N = 3. Data are presented as mean ± SD. **P < 0.01, ***P < 0.001, compared with the controls

α-mangostin (CAT: M3824, purity > 98%, Fig. 1A) was purchased from Sigma-Aldrich (St. Louis, MO, USA). Dulbecco’s modified Eagle’s medium, alpha modification of Eagle’s medium (α-MEM), fetal bovine serum (FBS), and penicillin/streptomycin were purchased from Gibco-BRL (Gaithersburg, MD, USA). Recombinant mouse macrophage colony-stimulating factor (M-CSF) and RANKL were acquired from R&D Systems (Minneapolis, MN, USA). The specific primary antibodies obtained from Cell Signaling Technology (Danvers, MA, USA) were as follows: c-Fos (#2250), NFATc1 (#8032), β-tubulin (#2146), phospho-JNK (#4668), IκBα (#4814), phospho-IκBα (#2859), p65 (#8242), phospho-p65 (#3033), phospho-IκB kinase (IKK)α/β (#2697), IKKβ (#8943), extracellular signal-related kinase (ERK) (#4695), phospho-ERK (#4370), JNK 1/2 (#9252), p38 (#9212), phospho-p38 (#4511), tartrate-resistant acid phosphatase (TRAP) (#92345), Human-reactive STING pathway antibody sampler kit (#38866). The cathepsin K (CTSK) antibody (ab37259) was obtained from Abcam (Cambridge, UK). TRAP staining kits, berberine, and anisomycin were purchased from Sigma-Aldrich.

### Bone marrow‐derived macrophage isolation and osteoclast differentiation

PBS and DMSO (Sigma-Aldrich) were used as vehicle and negative control for all treatments. Bone marrow cells were acquired from the long bones of 6-week-old male C57BL/6 mice, as described previously [22], and the bone marrow cells in femurs and tibias were flushed out and cultured in α-MEM supplemented with 10% FBS, 100 U/mL penicillin, 100 µg/mL streptomycin, and 25 ng/mL M‐CSF at 37 °C for 5 days to differentiate into bone marrow-derived macrophages (BMMs). Next, cells were respectively seeded into 48-well plates (~ 1 × 10^4^ cells/well in triplicate) and treated with different concentrations of α-mangostin (0, 0.5, 1, or 2 μmol/L) in the presence of 25 ng/mL M-CSF and 50 ng/mL RANKL. The culture medium was replaced every 2 days. After culturing for 5 days, the cells were fixed with 4% paraformaldehyde (PFA) and then stained for TRAP based on the instructions. TRAP‐positive multinucleated cells with ≥ 3 nuclei were counted using a light microscope (BX51; Olympus, Tokyo, Japan).

### Cell viability assay

To test the effects of α-mangostin on BMM viability, Cell Counting Kit 8 (CCK8) assays were performed. Based on the manufacturer’s instructions (Dojindo, Shanghai, China), cells were seeded in 96-well plates (8 × 10^3^ cells per well) and cultured in complete α-MEM medium for 48 or 96 h with different concentrations of α-mangostin (0–4 µM). Next, CCK8 reagent (10 µL) was added to each well, and the plate was incubated for another 2–4 h. The optical density (OD) was measured using a MR7000 microplate reader (Dynatech, Melville, NY, USA) at 450 nm. The viabilities of BMMs exposed to α-mangostin are expressed as a percentage of untreated cells.

### Constructing stable overexpression cell lines (transfection)

To validate that ACP5 gene was involved in α-mangostin inhibitory on osteoclagenesis. An ACP5 gene overexpression plasmid and negative control (NC) plasmid were purchased from Genepharma Corporation (Shanghai, China). All the procedures were followed with the manufacture protocols. The transfected cells were treated with 2 μg/mL puromycin until all the cells in the control group died (untransfected cells). The transfection efficiency of the cells was further confirmed by western blot before they were used in experiments.

### Hoechst 33342 staining

Cells (4 × 10^5^ cells per well) were placed in six-well plates and treated with different concentrations of α-mangostin for 5 days with the medium changed every 2 days, then cells were incubated with Hoechst 33342 for 20 min. A fluorescence microscope (Olympus) was utilized to visualize morphological changes of apoptotic cells at 365 nm.

### Apoptosis flow cytometry assay

Apoptosis was measured using Annexin V-fluorescein isothiocyanate/propidium iodide (FITC/PI) apoptosis kits (Multi-Sciences, Hangzhou, Zhejiang, China). BMMs (2 × 10^5^ per well) were seeded in six-well plates and cultured with different concentrations of α-mangostin (0, 0.5, 1, 2 μmol/L) for 24 h. After washing the cells with phosphate-buffered saline (PBS), they were collected and incubated with the reagents for 20 min in the dark. All steps were carried out according to the manufacturer’s protocols. V-FITC (+)/PI (−) and Annexin V- FITC (+)/PI (+) staining signified cells that were in the early and later stages of apoptosis, respectively. All the samples were analyzed by a flow cytometer (FACSCalibur, BD Biosciences, Franklin Lakes, NJ, USA).

### In vitro osteoclast differentiation

BMMs were planted into a 96-well plate at a density of 8 × 10^3^ cells per well, and cultured in complete α-MEM supplemented with 25 ng mL × 1 M-CSF, 50 ng mL × 1 RANKL, and different concentrations of α-mangostin (0, 0.5, 1, and 2 µM). The culture medium was replaced every 2 days. After 5 days, the cells were washed twice with PBS, fixed with 4% PFA, and stained for TRAP. TRAP-positive cells with ≥ 3 nuclei were counted as mature osteoclasts under a light microscope.

### RNA extraction and quantitative polymerase chain reaction (PCR)

Total RNA from cultured cells was extracted using the TRIzol reagent (Invitrogen, Carlsbad, CA, USA) according to the manufacturer’s protocols. Complementary DNA was synthesized from 1 µg of total RNA using PrimeScript RT Master Mix (TaKaRa Biotechnology, Otsu, Japan). Real-time PCR was performed using the TB Green Premix Ex Taq kit (TaKaRa Biotechnology) on a StepOnePlus Real-Time PCR System (Applied Biosystems, Foster City, CA, USA). Each reaction was run at the following conditions: 95 °C for 60 s and then 40 cycles of 95 °C for 10 s, 60 °C for 20 s and 72 °C for 20 s. Glyceraldehyde 3-phosphate dehydrogenase (GAPDH) served as the endogenous control. The mouse primer sequences are shown in Table 1.

**Table 1:** Primers used for quantitative PCR

Gene	Forward (F) and reverse (R) primer sequence (5′–3′)
GAPDH	F: ACCCAGAAGACTGTGGATGG R: CACATTGGGGGTAGGAACAC
CTSK	F: CTTCCAATACGTGCAGCAGA R: TCTTCAGGGCTTTCTCGTTC
TRAP	F: CTGGAGTGCACGATGCCAGCGACA R: TCCGTGCTCGGCGATGGACCAGA
DC‐STAMP	F: AAAACCCTTGGGCTGTTCTT R: AATCATGGACGACTCCTTGG
c‐Fos	F: CCAGTCAAGAGCATCAGCAA R: AAGTAGTGCAGCCCGGAGTA
NFATc1	F: CCGTTGCTTCCAGAAAATAACA R: TGTGGGATGTGAACTCGGAA
CTR	F: TGGTTGAGGTTGTGCCCA R: CTCGTGGGTTTGCCTCATC

### F-actin ring immunofluorescence and resorption pit assays

In order to visualize F-actin rings, BMMs were treated with 25 ng/mL M-CSF and 50 ng/mL RANKL for 4 days. We seeded differentiated osteoclasts (2 × 10^3^ cells/cm^2^) onto bovine bone slices and allowed them to adhere overnight. Then cells were treated with different concentrations of α-mangostin (0, 0.5, 1, or 2 µM) for another 2 days. The cells were then fixed with 4% PFA for 15 min, permeabilized with 0.4% Triton X-100 for 10 min, then stained with rhodamine-conjugated phalloidin (1:200; Invitrogen) diluted in 0.5% bovine serum albumin (BSA)–PBS for 30 min. Fluorescent images were captured utilizing a fluorescence microscope (EU5888, Leica, Wetzlar, Germany) and analyzed by ImageJ software [National Institutes of Health (NIH), Bethesda, MD, USA]. To observe resorption pits, the bone slices were washed twice with PBS, and adhered cells were removed by mechanical brushing. Bone slice images were acquired with a scanning electron microscope (SEM; S-4800, Hitachi, Japan) and analyzed by ImageJ software.

### Western blotting

The main signaling pathways affected by α-mangostin were detected by western blotting. Cells were treated with or without 2 µM α-mangostin for 4 h, then stimulated with 50 ng mL × 1 RANKL for 0, 5, 10, 20, 30, or 60 min. To explore the effects of α-mangostin on c-Fos and NFATc1 expression, BMMs were seeded in six-well plates (1 × 10^5^ cells per well) and cultured with 25 ng mL × 1 M-CSF and 50 ng mL × 1 RANKL in the presence or absence of 2 µM α-mangostin for 0, 2, 4, or 6 days. Then the cells were collected and lysed with radioimmunoprecipitation assay buffer (Sigma-Aldrich) containing a protease inhibitor and phosphatase inhibitor cocktail (Sigma-Aldrich). According to the steps in the manufacturer’s protocols, the supernatant was collected. Bicinchoninic acid protein assay kits (Beyotime, Shanghai, China) were used to quantify the total amounts of protein. Equal amounts of protein samples were separated by 8–15% sodium dodecyl sulfate–polyacrylamide gel electrophoresis at 75 V for 1.5 h and subsequently transferred onto 2.2-µm polyvinylidene fluoride membranes (Millipore, Burlington, MA, USA) at 250 mA for 2 h in a humid atmosphere. The membranes were blocked with 10% milk or 5% BSA (Sigma-Aldrich) and incubated with the primary antibody overnight at 4 °C. After three washes in Tris-buffered saline with Tween 20 (10 min each), the membranes were incubated with horseradish peroxidase-conjugated secondary antibodies (Huabio, Hangzhou, Zhejiang, China) for 1 h at room temperature. The target bands were developed with enhanced chemiluminescence kits (Millipore).

### Mouse model of LPS‐induced calvarial osteolysis

All animal care and experimental protocols were designed and performed in compliance with the NIH Guide for the Care and Use of Laboratory Animals and the Guide of the Animal Care Committee of Zhejiang University. Thirty 6‐week‐old male C57BL/6 mice weighing 18–22 g was purchased from Experimental Animal Center of Zhejiang University. A mouse model of LPS‐induced calvarial osteolysis was established, as described previously, to explore the effects of α-mangostin on inflammatory bone loss in vivo. After acclimatizing to the laboratory for 1 week, mice were randomly divided into the following three experimental groups (n = 5 each): sham, LPS (vehicle), and LPS + α-mangostin groups (both LPS and α-mangostin were first dissolved in dimethyl sulfoxide and then diluted with PBS). After anesthetization with intraperitoneal sodium pentobarbital (50 mg/kg), the cranial periosteum of mice was separated, and 5 mg/kg body weight (50 μl 10 mM) LPS (Sigma‐Aldrich) in PBS was embedded under the periosteum at the middle suture of the calvaria in the LPS (vehicle) and LPS + (100 μl 5 mM) α-mangostin groups on days 1 and 4, while PBS was injected in the sham group. Mice in the LPS + α-mangostin group also received daily subcutaneous injections of 10 mg/kg α-mangostin for 7 days. Mice in the sham and LPS groups were administered PBS as a control. Α-mangostin doses were determined according to previous studies [17, 27]. All the mice were sacrificed on day 7, and their calvaria were harvested for subsequent analysis.

### Micro-computed tomography (CT) scanning

Calvaria were measured (n = 5 per group) by a high-resolution micro-CT (Skyscan 1072, Aartselaar, Belgium). The scanning protocol was set at an isometric resolution of 9 µm and X-ray energy settings of 80 kV and 80 µA. Then, three-dimensional reconstruction was performed, and a 3 mm × 3 mm region of interest surrounding the midline suture was selected for further qualitative and quantitative analyses. Bone volume/tissue volume (BV/TV), number of porosities, and porosity percentage for each specimen were measured as reported previously [23].

### Hematoxylin and eosin (H&E) and TRAP staining

Fixed tissue samples (n = 5 per group) were decalcified in 10% ethylenediaminetetraacetic acid (pH = 7.4) for 2 weeks and then embedded in paraffin. Next, the calvaria were cut into 4-μm-thick histological sections for H&E and TRAP staining. The sections were photographed under a light microscope (TE2000-S; Nikon, Tokyo, Japan). Histomorphometric parameters of BV/TV, erosion area, the number of TRAP-positive osteoclasts, and surface area of osteoclasts per bone surface (OcS/BS) were assessed for each sample.

### Statistical analysis

All data are expressed as mean ± standard deviation (SD). Each experiment was repeated at least three times, and the results were analyzed with Prism 6.01 (GraphPad Software, San Diego, CA, USA). Two-tailed, unpaired Student’s t-tests were used for the comparisons between two groups. One-way analyses of variance with post hoc Newman–Keuls test were performed to analyze differences in multiple comparisons. Differences were considered significant at P < 0.05.

## Results

### Preventive effect of α-mangostin on RANKL-induced osteoclast differentiation in vitro

**Fig. 2:**
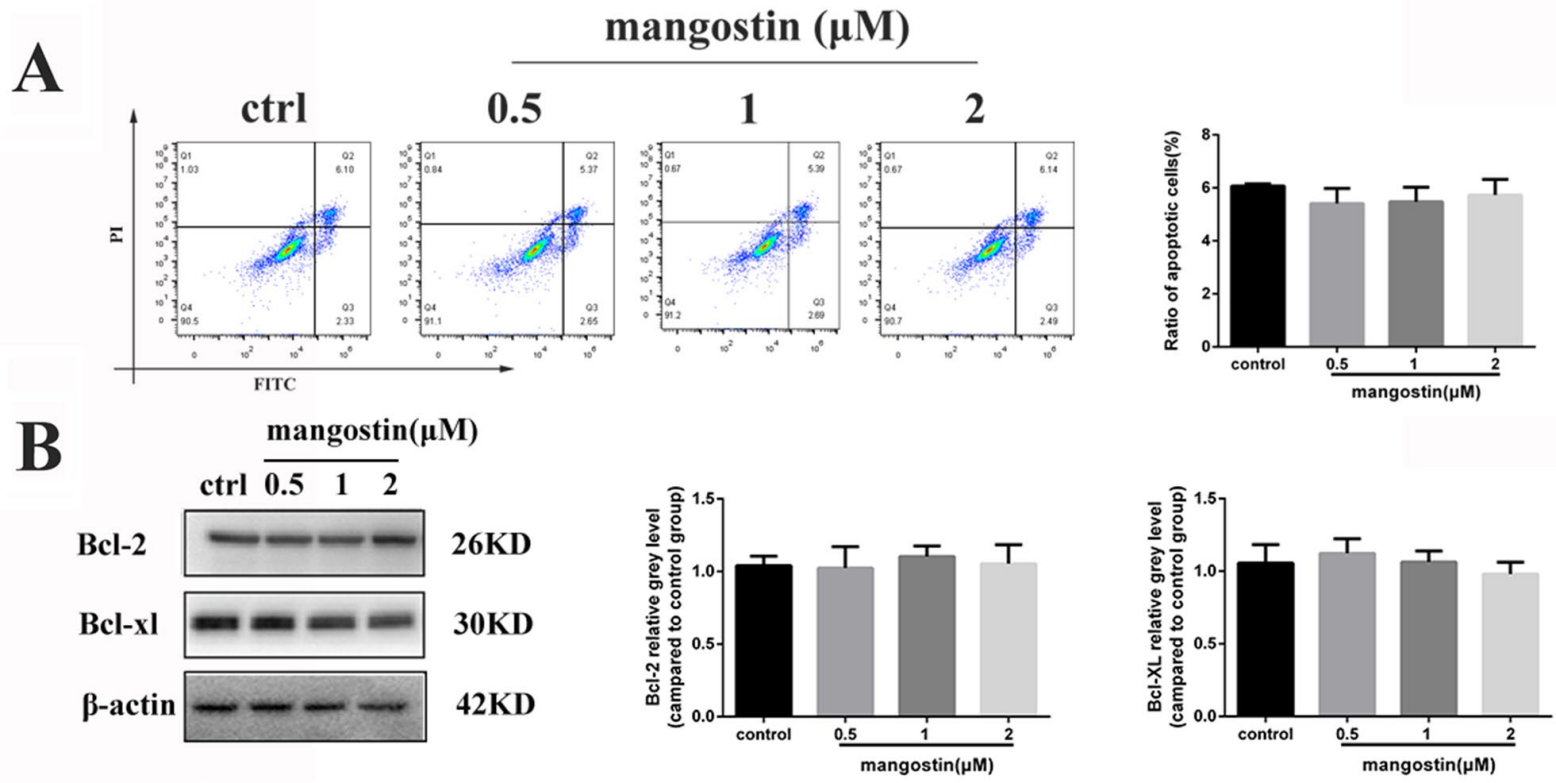
α-mangostin does not induce the apoptosis of BMMs in vitro. **A** BMMs were treated with different concentrations of α-mangostin for 5 days, and the level of apoptosis was measured by flow cytometry, N = 3. **B** The expression of those apoptosis-related proteins, which including BCL-2 and BCL-XL, was measured by a Western blot assay, N = 3. Error bar = mean ± SD

Cell viability assays were performed to evaluate a potential cytotoxic effect of mangostin on BMMs. The cells were treated with different concentrations of mangostin (0, 0.5, 1, 2, 4 µmol/L) for 48 or 96 h. The CCK-8 results revealed that mangostin caused no obvious BMM cytotoxicity at concentrations ≤ 2 μmol/L (Fig. 1B). Flow cytometry showed that mangostin administered at a concentration of 0–2 μmol/L did not cause BMM apoptosis, which was also confirmed by the absence of significant changes in apoptosis-related proteins (Fig. 2). When the concentration of mangostin reached 4 μM, the flow cytometry results indicated that 64.94% of BMMs were apoptotic. In addition, Hoechst 33342 staining, as a classic method for observing apoptotic cell nucleus shrinkage, also showed obvious cell apoptosis under 4 μM α-mangostin. There were also corresponding changes in apoptotic protein expression levels (Additional file 1: Fig. S1). Based on these results, we choose 2 μmol/L as the highest concentration for subsequent experiments.

To explore the preventive effect of α-mangostin against osteoclastogenesis, BMMs were treated with different concentrations of α-mangostin (0, 0.5, 1, 2 μmol/L) in the presence of M-CSF (25 ng/mL) and RANKL (50 ng/mL). After 5 days of incubation, the osteoclasts had differentiated into BMMs as confirmed by TRAP staining. Notably, α-mangostin reduced osteoclast differentiation in a dose-dependent manner (Fig. 1C and D). There were numerous mature TRAP-positive multinucleated osteoclasts in the control group, but the number and area of osteoclasts were significantly decreased in a dose-dependent manner in the α-mangostin groups. Next, α-mangostin was added at different stages of osteoclast formation to clarify which stage of osteoclast formation is affected by α-mangostin. Specifically, cells were treated with 2 µM α-mangostin at early (day 1–2), middle (day 3–4), late (day 5–6), and final stage (day 1–6). Compared with the other groups, the numbers and sizes of osteoclasts were significantly reduced in the final stage and early-stage groups (Fig. 1E and F). Correspondingly, there was a slight decrease in the middle stage group, but this inhibition effect was not significant in the late stage α-mangostin group. Finally, we explored whether the inhibitory effect of α-mangostin would be offset in BMMs overexpressing the TRAP gene (ACP5). We constructed stable transfected cells overexpressing ACP5 (S2A), and again evaluated osteoclast numbers and sizes. Despite the presence of α-mangostin, osteoclasts overexpressing ACP5 were able to regain the physiological function of bone resorption. This result confirmed TRAP as a key pathway for α-mangostin to hamper osteoclastogenesis. After overexpression of ACP5, despite the inhibitory effect of α-mangostin, BMMs could still be induced to differentiate into osteoclasts, but the sizes and numbers were reduced compared to the control group (Additional file 1: Fig. S1D4). Overall, these data demonstrate that α-mangostin had a suppressive effect on osteoclast formation, especially at the early stage of differentiation.

### α-mangostin decreases RANKL‐stimulated osteoclast‐specific gene expression

**Fig. 3:**
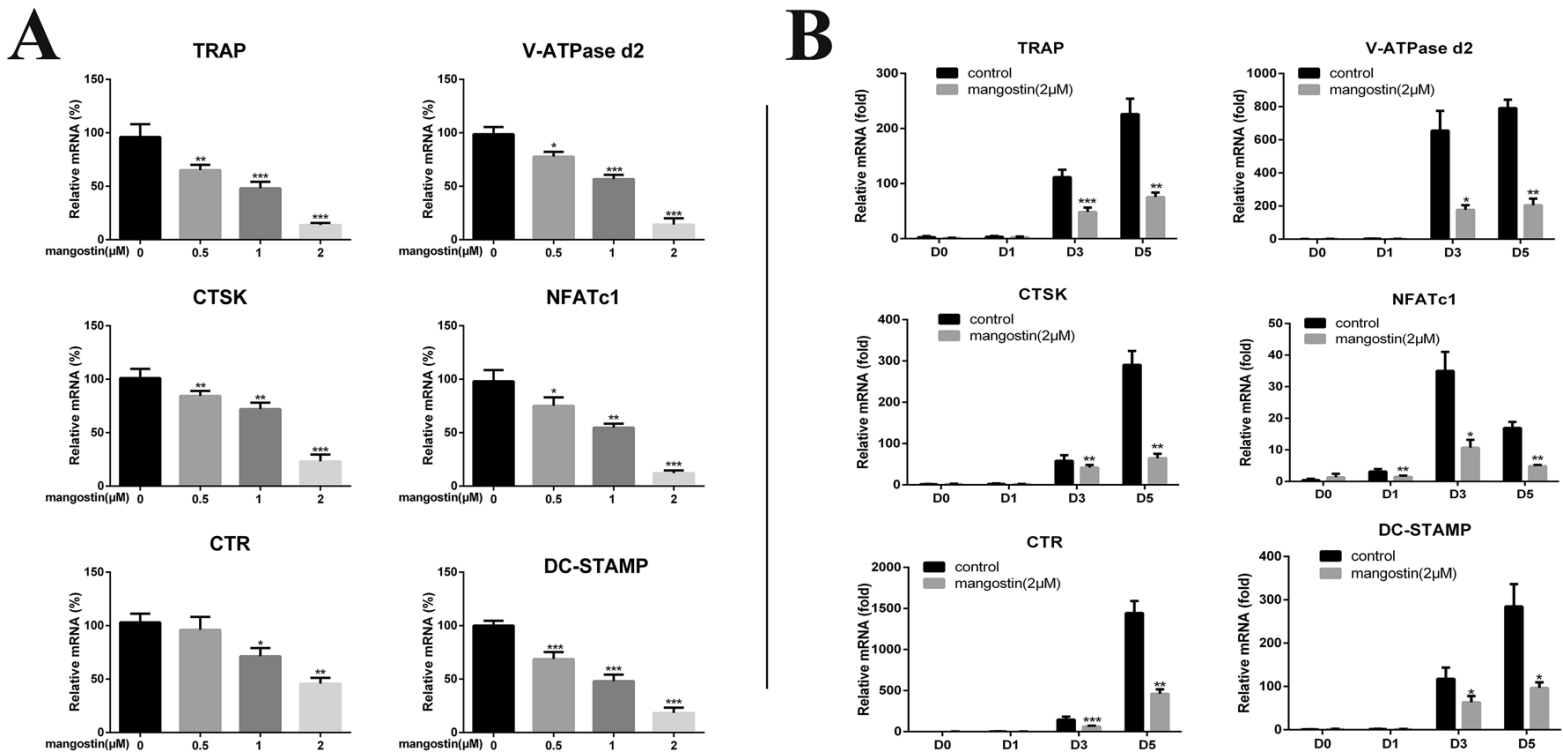
α-mangostin suppresses RANKL‐induced expression of osteoclast‐related genes. **A** BMMs were treated with M‐CSF, RANKL and different concentrations of α-mangostin for 5 days, N = 3. **B** BMMs were cultured with M‐CSF (25 ng/mL) and RANKL (50 ng/mL), with or without 2 μmol/L α-mangostin, for 0, 1, 3 or 5 days. The mRNA expression of those osteoclast‐related genes, including TRAP, CTSK, CTR, V-ATPase d2, NFATc1, DC‐STAMP, was determined by qPCR, N = 3. Data are shown as mean ± SD. *P < 0.05, **P < 0.01, ***P < 0.001, compared with the controls

Next, we investigated the effect of α-mangostin on RANKL‐stimulated osteoclast‐specific gene expression. After pretreatment with α-mangostin at concentrations of 0, 0.5, 1, or 2 μmol/L for 4 h, BMMs were stimulated by RANKL (50 ng/mL) for 48 h. The RT-PCR results confirmed that α-mangostin suppressed the expression levels of osteoclast-related genes including Ctsk, Dc-stamp, TRAP, CTR, V-ATPase-d2, and NFATc1 in time-dependent (Fig. 3A) and dose-dependent manners (Fig. 3B). These data showed that α-mangostin could inhibit osteoclastogenesis.

### Preventive effect of α-mangostin on bone resorption in vitro

**Fig. 4:**
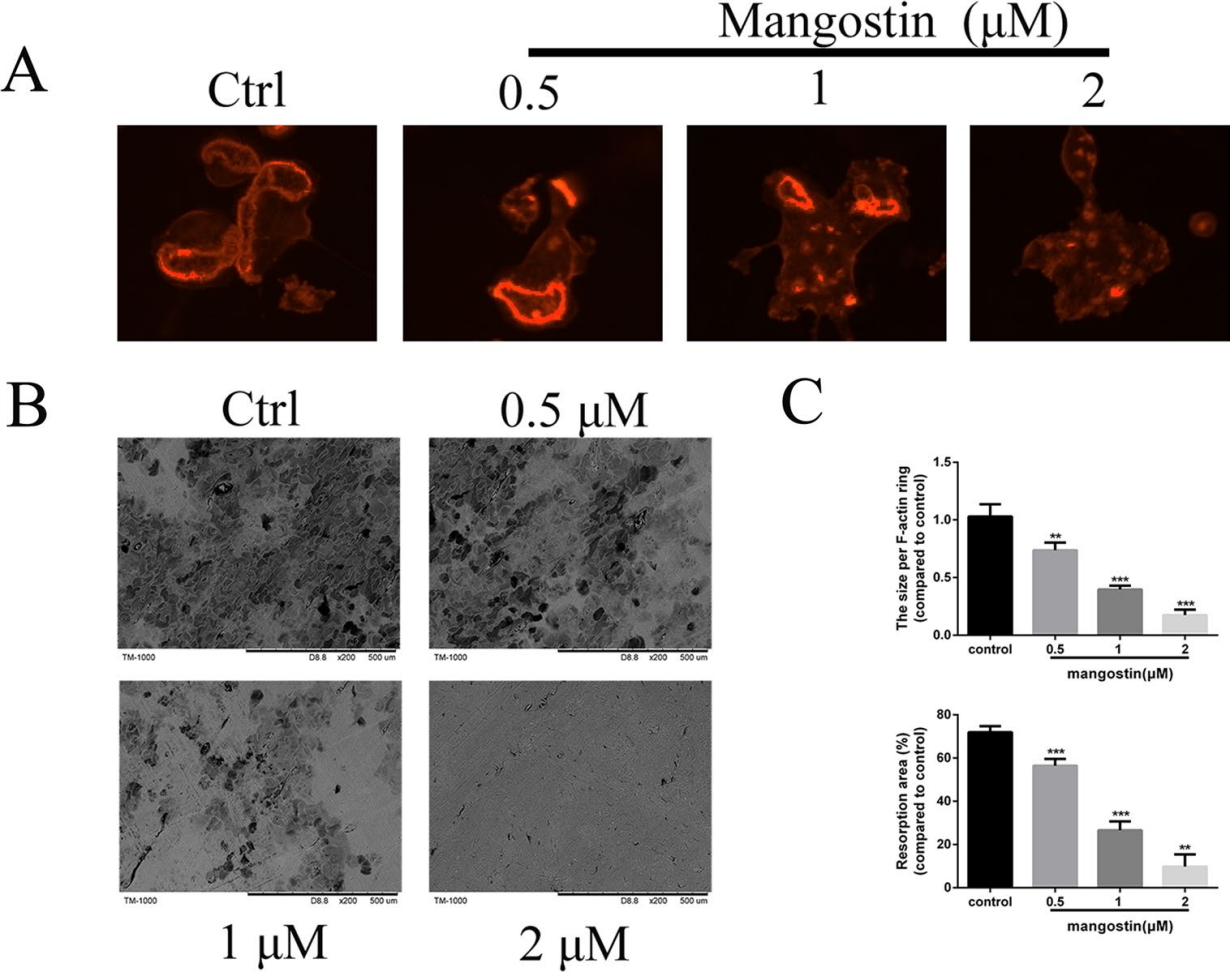
α-mangostin attenuates bone resorptive activity of mature osteoclasts and inhibits F‐actin ring formation in vitro. Same number of BMM‐derived mature osteoclasts were seeded onto bovine bone slices and were treated with the indicated α-mangostin concentrations for 2 days. **A** The cells were stained with rhodamine‐conjugated phalloidin and representative fluorescence microscope images of F‐actin rings.are shown. scale bar = 20 μm. **B** Representative images of bone resorption pits were acquired by scanning electron microscopy (SEM); scale bar = 100 μm. **C** The resorption area of bone discs and the size of F-actin rings were quantified using the ImageJ software, N = 3. Error bar = mean ± SD. **P < 0.01, ***P < 0.001, compared with the controls

α-mangostin was previously shown to inhibit osteoclast formation, so we wondered if it could also inhibit their function. BMMs (1 × 10^4^ cells/well) were seeded on Osteo Assay Plates and then incubated with RANKL and M-CSF and different concentrations of α-mangostin for 5–6 days. The tight F-actin ring is necessary for osteoclasts to perform bone resorption and can be used to evaluate whether mature osteoclasts are functioning. The immunofluorescence results showed that the shape and size of the F-actin ring were destroyed by α-mangostin in a dose-dependent fashion (Fig. 4A). Extensive bone resorption areas and larger pits were observed in the control group, while α-mangostin treatment significantly decreased the pit area, and almost no resorption pits were found on bone slices treated with 2 µM α-mangostin (Fig. 4B and C). Similarly, we verified the effect of ACP5 overexpression on α-mangostin’s inhibition of osteoclast function. Despite the presence of α-mangostin, osteoclasts overexpressing ACP5 can regain the physiological function of bone resorption (Additional file 2: Figure S2B3). Together, our results indicate that α-mangostin suppresses the F‐actin ring formation and bone resorptive activity of mature osteoclasts in vitro.

### α-mangostin inhibits RANKL-induced NF-κB and MAPK signaling

**Fig. 5:**
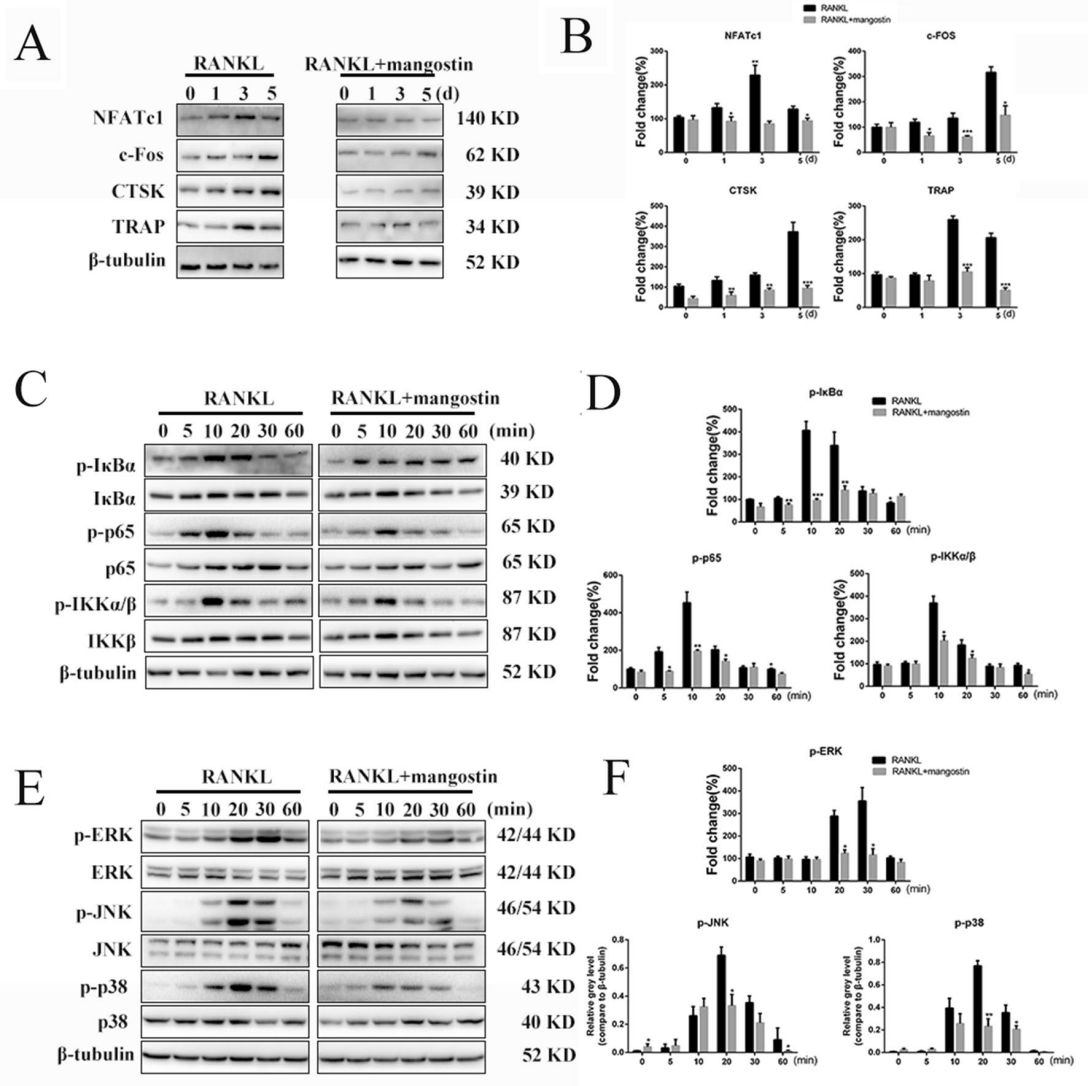
α-mangostin inhibits osteoclastogenesis by specifically suppressing RANKL-induced phosphorylation of NF-κB/MAPK signaling pathways. **A** The protein expression levels of NFATc1, c-Fos, TRAP and CTSK in BMMs treated with 50 ng/mL RANKL with or without 2 µM α-mangostin for 0, 1, 3, or 5 days, N = 3. **B** The expression of these proteins were quantified using the ImageJ software, N = 3. **C**, **E** BMMs were pretreated with or without 2 µM α-mangostin for 4 h and then cultured with RANKL for the indicated periods. **D**, **F** The gray levels of phosphorylated p65, ERK, JNK, and p38 were quantified and normalized relative to their total protein counterparts. The gray levels of p-IκBα and IκBα were normalized to β-tubulin, N = 3. Data are presented as mean ± SD. *P < 0.05, **P < 0.01, ***P < 0.001, compared with RANKL alone

First, we verified that α-mangostin could inhibit the expression of osteoclast-related proteins induced by RANKL in a dose-dependent manner (Fig. 5A and B). Due to the presence of RANKL in the control group, the osteoclast-related proteins NFATc1, c-Fos, CTSK, and TRAP were significantly increased, while α-mangostin obviously inhibited their expression, especially 3–5 days after adding RANKL.

NF‐κB signaling is regarded as having a key role in osteoclast differentiation, so we wondered whether the effect of α-mangostin on RANKL‐stimulated osteoclastogenesis was regulated via NF‐κB activation [5]. In this study, the phosphorylation and protein levels of NF‐κB p65 and IκBα were measured by western blot. Before 1 h stimulation with 50 ng/mL RANKL, the BMMs were preincubated with different concentration of α-mangostin for 4 h. Cells treated with α-mangostin showed less phosphorylation of NF‐κB p65 and IκBα, and degradation of IκBα protein was also significantly decreased (Fig. 5C and D). In addition, MAPKs (ERK, JNK, and p38) were phosphorylated by stimulation with RANKL, while α-mangostin treatment significantly suppressed the phosphorylation levels of these proteins (Fig. 5E), which was confirmed by the quantitative analysis (Fig. 5F). We used the JNK agonist anisomycin to carry out the reverse experiment. The results suggested that α-mangostin indeed inhibited JNK pathway activation, but the ability of α-mangostin to attenuate osteoclast formation and function was not completely eliminated (Additional file 1: Fig. S1D5 and Additional file 2: S2B4, C). These results revealed that α-mangostin inhibits RANKL‐induced activation of NF‐κB and MAPK signaling in vitro.

### Effect of α-mangostin on LPS‐induced osteolysis in a mouse calvarial model

**Fig. 6:**
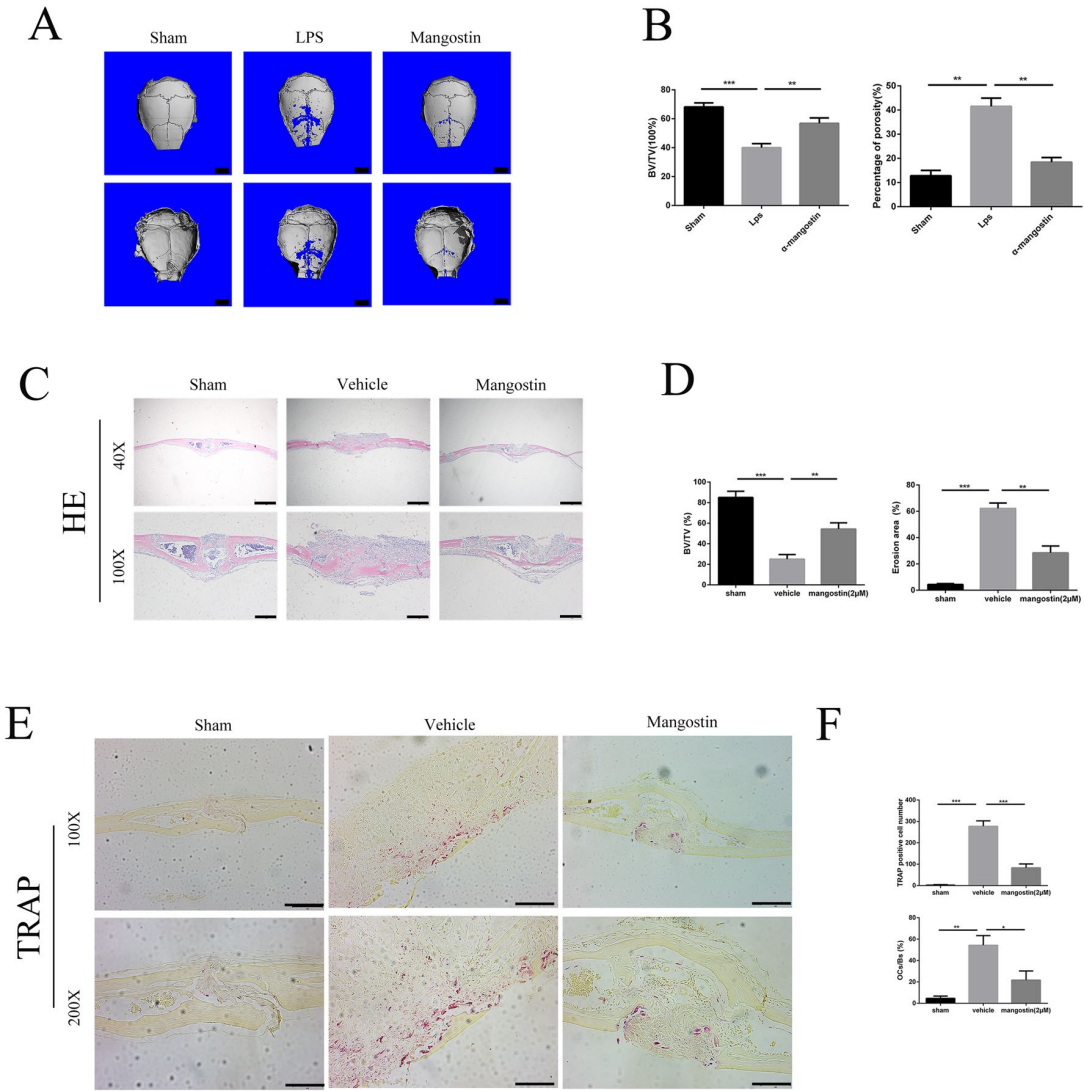
α-mangostin prevents LPS‐induced bone loss inhibiting osteoclast activity in a mouse calvarial model. **A** Representative micro-CT reconstruction images of the calvarial each group. Scale bars = 1 mm. **B** Quantitative analysis of the bone volume against tissue volume (BV/TV, %) and the percentage of porosity (%) was performed using the micro‐CT data, N = 5. **C** Representative images of H&E staining of calvarial bone sections from sham, vehicle, and 2 µM α-mangostin-treated groups. Scale bars = 200 µm. **D** Histomorphometric analysis of the BV/TV and erosion area was performed, N = 5. **E** Representative images of TRAP staining of calvarial bone sections from three groups; cale bar = 50 μm. **F** Quantitative analysis of the number of TRAP-positive osteoclasts, and the percentage of osteoclast surface per bone surface (OcS/BS, %) were performed, N = 5. *P < 0.05 and **P < 0.01, ***P < 0.001, compared with the mice in the vehicle group

To investigate effects of α-mangostin on pathological osteolysis in vivo, an LPS-induced murine calvarial osteolysis model was established. LPS was embedded under the periosteum at the middle suture of the calvaria with or without α-mangostin (10 mg/kg) in the LPS (vehicle) and LPS + α-mangostin groups. Mice in the LPS + α-mangostin group were intragastrically administered 10 mg/kg α-mangostin every day for 7 days. Mice in the sham and LPS (vehicle) groups were administered PBS intragastrically as a control. After 7 days, the calvaria were collected and fixed in 4% PFA, then analyzed by micro-CT and histology (Fig. 6A). Micro‐CT images showed that calvaria bone loss was decreased by α-mangostin treatment, while extensive bone erosion was observed in the LPS (vehicle) group compared with the sham group. Quantitative analysis indicated that decline of BV/TV and increased porosity induced by LPS were all attenuated by α-mangostin treatment (Fig. 6B).

Histological assessments further confirmed the results from Micro‐CT showing that α-mangostin had a therapeutic effect against osteolysis. H&E staining showed that LPS induced severe osteolytic changes in the LPS (vehicle) group, but a significantly reduced extent of bone erosion was found in the α-mangostin group (Fig. 6C). Moreover, the number of osteoclasts was decreased in the α-mangostin group compared with LPS (vehicle) group as shown by TRAP staining (Fig. 6E). Finally, we extracted protein samples from calvaria tissue from each group to examine the expression of pathway-related proteins. Similar to previous results, the expression levels of these proteins were significantly inhibited by mangosteen. These data suggest that α-mangostin hindered LPS-induced osteolytic bone loss in vivo.

## Discussion

Increased osteoclast-induced bone resorption is an important factor leading to periprosthetic osteolysis and osteoporosis [2, 24]. The balance between osteoblast‐mediated bone formation and osteoclast‐mediated bone resorption is critical for appropriate bone turnover and remodeling, so it is important to identify strategies to treat bone diseases [5]. In our study, we demonstrated for the first time that α-mangostin could inhibit RANKL‐induced osteoclastogenesis by inhibiting NF‐κB and MAPK signaling in vitro and hinder LPS-induced osteolytic bone loss in a mouse calvarial model.

α-mangostin has extensive biological activities and pharmacological properties; it is antioxidant, antineoplastic, antiproliferation, and induces apoptosis [14, 25, 26]. A recent report described the use of α-mangostin in treating rheumatoid arthritis and a α-mangostin-loaded self-micro emulsion was designed [26]. Recently, it was reported that α-mangostin can block LPS-induced activation in RAW264.7 cells, thereby inhibiting the secretion of IL-1β, IL-6, NO, and cyclooxygenase 2 [27]. Α-mangostin was also reported to inhibit the activation of TAK1-NF‐κB to exert anti-inflammatory effects, which makes it a potential choice for treating inflammatory diseases [28]. Previous studies have shown that α-mangostin has an inhibitory effect on the osteoclast differentiation of RAW264.7 cells. However, it is more scientific to use mouse BMMs as the research object for in vitro experiments as the results are more credible. We also confirmed the role of α-mangostin in inhibiting osteoclasts at the animal level. Our current research confirms that α-mangostin has great potential for treating osteoporosis.

We examined whether α-mangostin had a toxic effect on BMMs and if it could inhibit the osteoclast differentiation. The CCK-8 results showed that α-mangostin had no obvious inhibitory effect on BMMs at concentrations < 2 μmol/L. To clarify whether the effect of α-mangostin on osteoclast formation is indeed achieved by inhibiting their differentiation rather than promoting apoptosis, we performed flow cytometry and western blotting, and the results were consistent with our previous conclusions. Additional file 1: Fig. S1 clearly shows that when the α-mangostin dose reached 4 μM, apoptosis-related protein expression was significantly increased. The flow cytometry results suggested that there was apoptosis in BMMs, and Hoechst staining further confirmed this conclusion. This was the basis to select the drug dosage for our follow-up studies. TRAP staining showed that at concentrations below cytotoxic levels, α-mangostin had a significant protective effect against RANKL-induced osteoclast differentiation. As the concentration of α-mangostin increased, the number of TRAP-positive cells decreased, and mature osteoclasts were rarely observed in the high concentration (2 μmol/L) treatment group. Based on the conclusions of previous studies of natural compounds, the inhibitory effect of berberine on osteoclast formation was used as our positive control [29]. To further determine when osteoclastogenesis was inhibited by α-mangostin, we added α-mangostin (2 μmol/L) at different stages of osteoclastogenesis. The experimental results were in line with our expectations. As shown in Fig. 1D, the earlier the α-mangostin intervention, the stronger the effect of obstructing osteoclast formation. Compared with the control group, osteoclastogenesis inhibition of α-mangostin was hardly seen in the late-stage group. Based on these results, we concluded that α-mangostin could inhibit osteoclast formation, especially in the early stage of osteoclastogenesis.

Under RANKL stimulation, the upregulation of the expression of several specific genes is closely related to osteoclast differentiation [8]. Real‐time PCR was then utilized to measure the inhibitory effect of α-mangostin on RANKL-induced mRNA expression of these genes (TRAP, NFATc1, CTSK, V-ATPase d2, CTR, and DC-stamp). As expected, α-mangostin blocked RANKL‐stimulated osteoclast-related gene expression in a dose- and time-dependent manner. This proved from another aspect that α-mangostin indeed suppressed osteoclast differentiation. After verifying the inhibitory effect of α-mangostin on osteoclast formation and RANKL-induced osteoclastic marker gene expression, we explored whether α-mangostin could also affect osteoclast bone resorption. The results showed marked bone resorption in the control group that was attenuated in the α-mangostin group, again demonstrating that α-mangostin hinders osteoclast function.

NF‐κB signaling is a classic pathway investigated in osteoclastogenesis studies [7]. When RANKL activates downstream signaling, the phosphorylation of NF-κBp65 and IκBα and the degradation of IκBα promote the activation and nuclear translocation of NF-κB p65, which will increase the expression of osteoclast‐specific genes and promote osteoclast formation and function [30, 31]. Based on this status quo, many researchers currently focus on pharmacological interventions for NF‐κB signaling related checkpoints. In our current study, we found that α-mangostin restricted the degradation of IκBα and phosphorylation of NF‐κB p65 and IκBα induced by RANKL stimulation. Furthermore, previous studies have shown that RANKL-induced osteoclastogenesis usually involves the activation of both NF-κB and MAPK signaling pathways. We therefore explored whether α-mangostin could also inhibit MAPK pathway activation. Not surprisingly, the phosphorylation of all three MAPK pathways (ERK, JNK, and p38) in RANKL-stimulated BMMs was blocked by α-mangostin at non‐cytotoxic concentrations.

Previous studies reported that JNK1 regulates RANKL-induced osteoclastogenesis by activating the Bcl-2-Beclin1-autophagy pathway, and our findings suggest that the JNK pathway is regulated by α-mangostin [32]. For this reason, we used the JNK activator anisomycin to explore whether α-mangostin-mediated inhibition of osteoclastogenesis could be reversed. The results confirmed that anisomycin indeed reversed the effect of α-mangostin on the JNK pathway, but the inhibitory effect of α-mangostin on osteoclast formation and function was not affected. This may be due to the fact that α-mangostin works through all three MAPK pathways, so simply altering one of them does not make a significant difference. When the LPS pathway is activated, TRAF-TBK1-IRF3 signaling also changes [33]. We used western blots to detect the expression of TBK1 and IRF3 proteins and found that α-mangostin did not affect the pathways affected by stimulation of RANKL. Therefore, we concluded that MAPK and NF-κB pathway activation was inhibited by α-mangostin, except for IRF3 (Additional file 2: Figure S2D).

Because previous results indicated that α-mangostin obstructed RANKL-induced osteoclast formation and decreased the expression of osteoclastic-related genes by blocking the NF-κB and MAPK signaling cascades in vitro, we investigated whether α-mangostin could inhibit pathological osteolysis in an LPS-induced murine calvarial osteolysis model [34]. Micro-CT scan results and histological examinations led us to conclude that α-mangostin reduced LPS-induced osteolysis in vivo. Regarding the choice of in vivo drug dosage and administration method, we referred to methods in previous studies [35, 36]. At first, we chose 20 mg/kg as the in vivo dose, but we observed inflammatory hyperplasia under the skin of the mouse skull. We the reduced the dose to 10 mg/kg, which is consistent with the effective dose in another study [37]. These results provide the first evidence that α-mangostin could be a potential treatment for osteoclast-related diseases.

Nevertheless, there are several limitations of the current study. First, because there is a balance between osteoclastic bone resorption and osteoblastic bone formation, further research on the effect of α-mangostin on osteoblasts is needed. The present study proved that α-mangostin markedly suppressed RANKL-induced osteoclast formation by inhibiting the pathway in vitro. We still need to study whether α-mangostin also exerts its anti-osteoclastogenic effect through this pathway in vivo. Unfortunately, we did not explore the exact binding target of α-mangostin. Identifying this target should explain why α-mangostin blocks NF-κB and MAPK signaling induced by RANKL. In the early stage of the RANKL pathway, after RANKL binds to RANK, tumor necrosis factor receptor-related factor 6 (TRAF6) is recruited to form a complex that further activates the MAPK and NF-κB pathways [38, 39]. Because the MAPK and NF-κB pathways share the same upstream promoter in TRAF6, it is reasonable to speculate that α-mangostin interferes in the binding process between TRAF6 and RANK.

## Conclusion

Our results demonstrate that α-mangostin inhibited RANKL-induced osteoclastogenesis and bone resorption in vitro. These inhibitory effects were mediated by suppressing RANKL-induced NF-κB and MAPK signaling. In addition, α-mangostin exerted a protective effect against LPS-induced inflammatory bone loss in vivo, indicating that it can be used as a potential drug to prevent or treat osteoclast-related diseases.

## Supplementary Information

**Additional file 1: Figure S1.** (A), When the concentration of α-mangostin is 4 μM, the proportion of apoptotic cells in the flow cytometry result. (B), The results of Hoechst 33342 staining indicated that there was no obvious cell apoptosis when the concentration of mangotin was 2 μM, but after reaching 4 μM, the proportion of apoptotic cells increased significantly. The red arrow means apoptotic cells. scale bar = 500 μm. (C), Expression of apoptosis-related proteins in BMMs treated with different concentrations of α-mangostin, N = 3. (D), The number and area of TRAP-positive cells were analyzed. 1: BMMs were treated with control medium. 2 and 3: BMMs were treated with α-mangostin and berberine. 4: BMMs (ACP5) were treated with α-mangostin. 5: BMMs were pretreated with anisomycin(5 ng/ml) and α-mangostin. scale bar = 200 μm. (E), The number and area of TRAP-positive cells were analyzed, N = 3. Data are presented as mean ± SD. **P < 0.01, compared with the controls; ***P < 0.001, compared with the treated groups.

**Additional file 2: Figure S2.** (A), Verify the effect of BMMs cell transfection at the protein level. (B), Representative images of bone resorption pits were acquired by scanning electron microscopy (SEM). 1: osteoclasts were treated with control medium. 2: osteoclasts were treated with α-mangostin. 3: osteoclasts differentiated from BMMs (ACP5) were treated with α-mangostin. 5: osteoclasts were pretreated with anisomycin(5 ng/ml) and α-mangostin. scale bar = 100 μm. (C), The expression level of p-JNK protein in the presence or absence of anisomycin and α-mangostin. (D), BMMs were pretreated with or without 2 µM α-mangostin for 4 h and then cultured with RANKL for the indicated periods. (E), Pathway-related proteins of each group were measured by western blot. Tissue protein is obtained from the skull by a tissue homogenizer.

## Data Availability

The data used to support the findings of this study are available from the corresponding author upon request.

## Abbreviations

α-MEM: Alpha modification of Eagle’s medium
BMM: Bone marrow-derived macrophages
CTSK: Cathepsin K
DMEM: Dulbecco’s modifed Eagle’s medium
DMSO: Dimethylsulfoxide
FACS: Fluorescence activating cell sorter
FITC: Fluorescein isothiocyanate
HE: Hematoxylin–eosin
HRP: Horseradish peroxidase
IHC: Immunohistochemistry
MAPK: Mitogenactivated protein kinase
M-CSF: Macrophage colony-stimulating factor
NC: Negative control
NF-κB: Nuclear factor-κB
OA: Osteoarthritis
PBS: Phosphate buffered saline
PI: Propidium iodide
RANKL: Receptor activators of NF-κB ligand
SD: Standard deviation
TRAF 6: Tumor necrosis factor receptor-related factor 6
TRAP: Tartrate resistant acid phosphatase
