# Supplementary figures and images for: α-Mangostin inhibits LPS-induced bone resorption by restricting osteoclastogenesis via NF-κB and MAPK signaling
Source: Chin Med. 2022 Mar 5;17:34. doi: 10.1186/s13020-022-00589-5 (PMC8898470; doi:10.1186/s13020-022-00589-5)

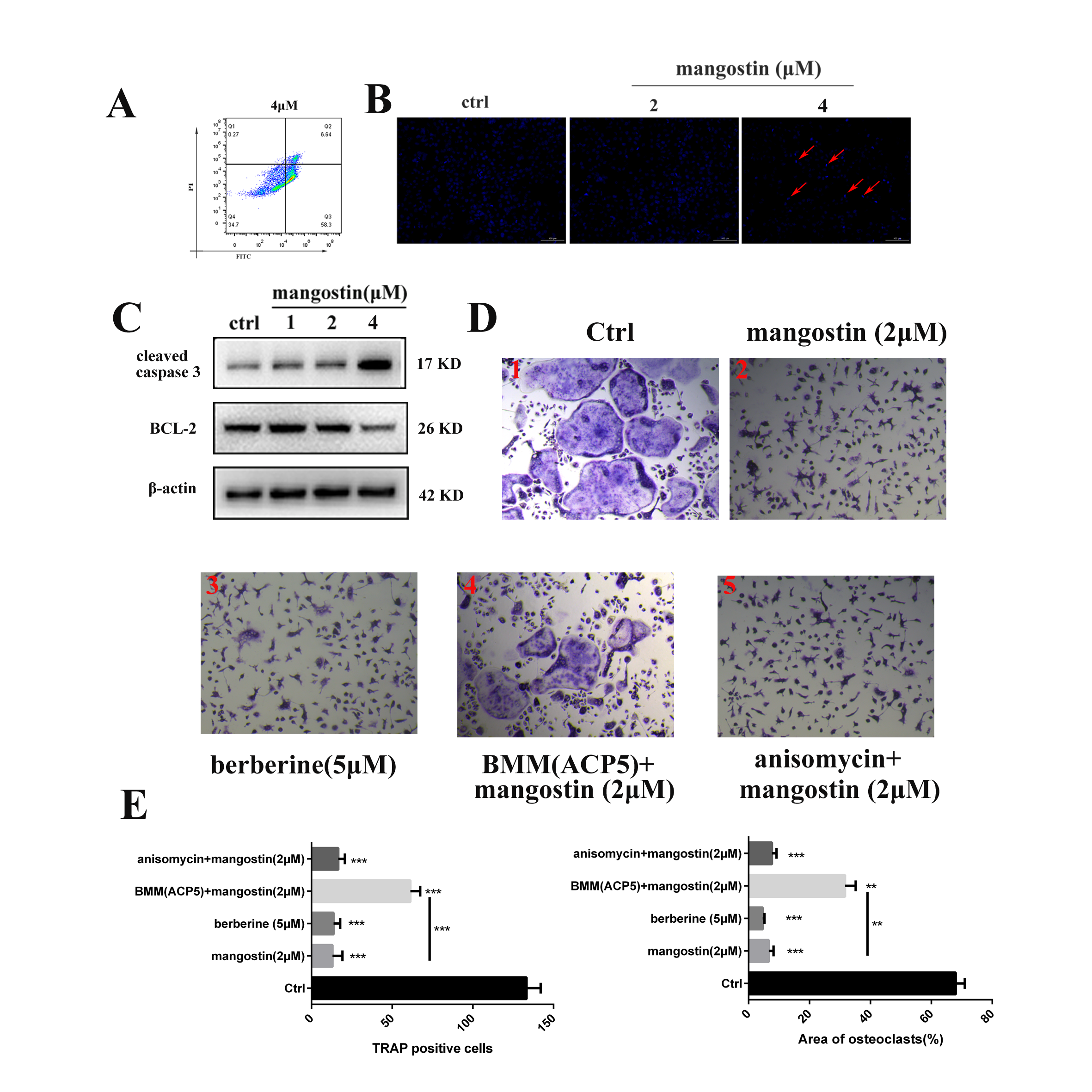

Supplement: Supplementary file 1 — Additional file 1: Figure S1. (A), When the concentration of α-mangostin is 4 μM, the proportion of apoptotic cells in the flow cytometry result. (B), The results of Hoechst 33342 staining indicated that there was no obvious cell apoptosis when the concentration of mangotin was 2 μM, but after reaching 4 μM, the proportion of apoptotic cells increased significantly. The red arrow means apoptotic cells. scale bar = 500 μm. (C), Expression of apoptosis-related proteins in BMMs treated with different concentrations of α-mangostin, N = 3. (D), The number and area of TRAP-positive cells were analyzed. 1: BMMs were treated with control medium. 2 and 3: BMMs were treated with α-mangostin and berberine. 4: BMMs (ACP5) were treated with α-mangostin. 5: BMMs were pretreated with anisomycin(5 ng/ml) and α-mangostin. scale bar = 200 μm. (E), The number and area of TRAP-positive cells were analyzed, N = 3. Data are presented as mean ± SD. **P < 0.01, compared with the controls; ***P < 0.001, compared with the treated groups. [file 13020_2022_589_MOESM1_ESM.tif]

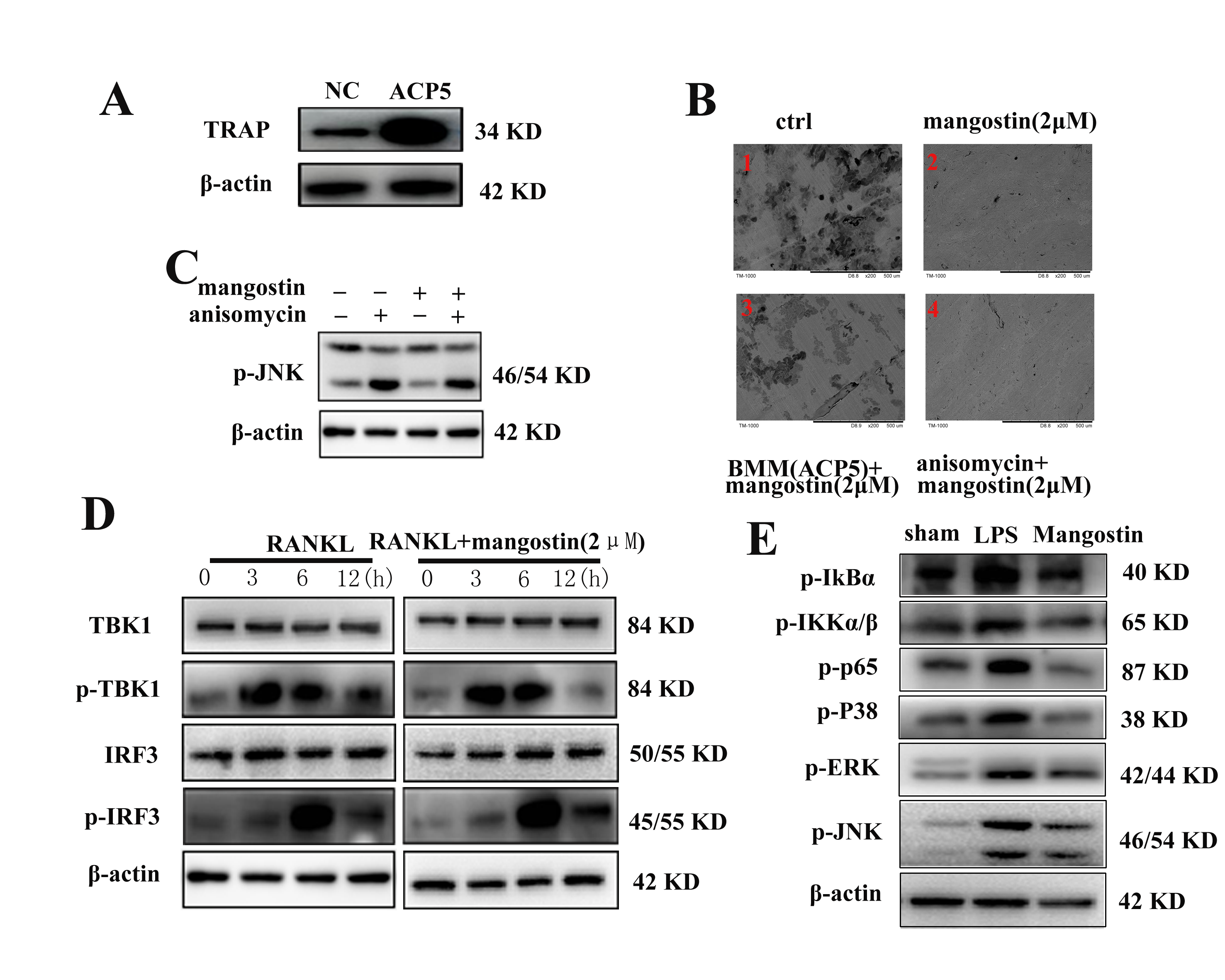

Supplement: Supplementary file 2 — Additional file 2: Figure S2. (A), Verify the effect of BMMs cell transfection at the protein level. (B), Representative images of bone resorption pits were acquired by scanning electron microscopy (SEM). 1: osteoclasts were treated with control medium. 2: osteoclasts were treated with α-mangostin. 3: osteoclasts differentiated from BMMs (ACP5) were treated with α-mangostin. 5: osteoclasts were pretreated with anisomycin(5 ng/ml) and α-mangostin. scale bar = 100 μm. (C), The expression level of p-JNK protein in the presence or absence of anisomycin and α-mangostin. (D), BMMs were pretreated with or without 2 µM α-mangostin for 4 h and then cultured with RANKL for the indicated periods. (E), Pathway-related proteins of each group were measured by western blot. Tissue protein is obtained from the skull by a tissue homogenizer. [file 13020_2022_589_MOESM2_ESM.tif]
